# Supplementary material for: Differential perovskite hemispherical photodetector for intelligent imaging and location tracking
Source: Nat Commun. 2024 Jan 17;15:577. doi: 10.1038/s41467-024-44857-4 (PMC10794423; doi:10.1038/s41467-024-44857-4)
Supplement: Supplementary file 1 — Supplementary Information [file 41467_2024_44857_MOESM1_ESM.pdf]

Supplementary Information for

Differential Perovskite Hemispherical Photodetector for Intelligent Imaging and Location Tracking

Xiaopeng Feng[1], Chenglong Li[1], Jinmei Song[1], Yuhong He[1], Wei Qu[1], Weijun Li[1], Keke Guo[1], Lulu Liu[1], Bai Yang[1,2], and Haotong Wei[1,2*]

[1]*State Key Laboratory of Supramolecular Structure and Materials, College of Chemistry, Jilin University, Changchun, 130012, P. R. China*

[2]*Optical Functional Theragnostic Joint Laboratory of Medicine and Chemistry, The First Hospital of Jilin University, Changchun, 130012 P.R. China*

*Email: hweichem@jlu.edu.cn

Differential Perovskite Hemispherical Photodetector for Intelligent Imaging and Location Tracking 1

Supplementary Notes...3

Materials 3

General Information 3

Supplementary Methods 4

Synthesis of N, N'-Bis(Boc)-1H-naphthalene-2-carboxamidine. 4

Synthesis of 1-(naphthalen-2-yl)guanidinium iodide. 5

Computational spectral reconstruction. 7

Supplementary Figures and Discussions 8

Synthesis and characterization of 1-(naphthalen-2-yl)guanidinium iodide............................ 8

The characterization of the 1-(naphthalen-2-yl)guanidinium iodide - Lead(II) iodide complex 11

The characterization of perovskite (with 1-(naphthalen-2-yl)guanidinium iodide) film 14

The photodetector performance. 20

The single-pixel Fast Fourier Transform imaging and the color classification..................... 25

The differential hemispherical photodetector for trace reconstruction 31

Supplementary References..40

Supplementary Notes

Materials

All chemicals were used without further purification. Lead (II) iodide (PbI_2, 99.99% powder), 2,9-dimethyl-4,7-diphenyl-1,10-Phenanthroline (BCP, >99% HPLC), Poly[bis(4-phenyl) (2,4,6-trimethylphenyl) amine (PTAA, Mw: 1000-10000 by GPC), C_{60} (99.5%) were purchased from Xi'an p-OLED. Methylamine hydrochloride (MACl, 98%), N, N-Dimethylformamide anhydrous (DMF, 99.9%), Acetonitrile anhydrous (ACN, 99.9%), 2-Methoxyethanol anhydrous (2-Me, 99.9%), Diisopropylethylamine (DIPEA, 99.5%), Trichloromethane iodide (TMSI, 98%), 2-naphthylamine (98%), N,N'-Bis(tert-butoxycarbonyl)-1H-pyrazole-1-carboxamidine (98%), Petroleum ether (boiling range: 30 ℃ ~ 60 ℃), ethyl acetate (AR, >99.5%), dichloromethane (AR, >99.5%), anhydrous methanol (MeOH, 99.99%), deuterated methanol (MeOD-d_4, 99%), deuterated acetonitrile (ACN-d_3, 99.8%) were purchased from Energy Chemical. L-ascorbic acid (L-AA, 99%) was purchased from Sigma Aldrich. Formamidine hydroiodide (FAI, 99%) was purchased from Great Cell Solar. Ether (AR) was purchased from Sinopharm Chemical Reagent.

General Information

EQE spectra were recorded under the illumination of monochromatic light from the Xenon lamp using a monochromator (Zolix) and detected by a computer-controlled Keithley 2400 SourceMeter. UV-visible absorption spectra were obtained using a Shimadzu 3600 UV-visible-NIR spectrophotometer. Photoluminescence spectra were obtained using Omni-λ3007i with a 375

nm laser. XRD data were collected using a PANalytical B.V.-Empyream Diffractometer with Cu Kα radiation. The SEM cross-section images were acquired by Hitachi Cold Field Emission SEM with Bruker X-ray. ^{1}H NMR and ^{13}C NMR spectra were obtained on Bruker AVANCE III 500 MHz. Chemical shifts were reported in ppm relative to the residual solvent peak (MeOD: ^{1}H, 3.34; ^{13}C, 49.03; ACN: ^{1}H, 1.96). High-resolution mass spectrometry data was obtained using Agilent 1290 - Bruker micrOTOF QII.

Supplementary Methods

Synthesis of N, N'-Bis(Boc)-1H-naphthalene-2-carboxamidine.

2-naphthylamine (1.43 g, 10 mmol) and N, N'-Bis(tert-butoxycarbonyl)-1H-pyrazole-1-carboxamidine (3.10 g, 10 mmol) were dissolved in 50 mL of dichloromethane, after which 1.804 g (10 mmol) diisopropylethylamine (DIPEA) was added to the mixture, which was protected by nitrogen and stirring at room temperature overnight. The mixed solution was extracted with 1% acetic acid aqueous solution and dichloromethane, and the organic layer solution was collected. The organic solvent is removed by evaporation and a white solid is obtained. The crude product was washed with 1% acetic acid solution and dried several times. The pure product was further purified by silica gel column chromatography: petroleum ether (PE): ethyl acetate (EA) = 9:1 (v/v) with a yield of 98% (3.78 g, 9.8 mmol). ^{1}H NMR ($CDCl_3$-*d*, 500 MHz): δ=11.72 (s, 1H), 10.55 (s, 1H), 8.20 (s, 1H), 7.79–7.84 (m, 3H), 7.67–7.70 (m, 1H), 7.50–7.40 (m, 2H), 1.58 (s, 9H), 1.55 (s, 9H)[1].

Synthesis of 1-(naphthalen-2-yl)guanidinium iodide.

N, N’-Bis(Boc)-1H-naphthalene-2-carboxamidine (3.85 g, 10 mmol) and trimethylsilane iodide (5 g, 25 mmol) were dissolved in 30 mL dichloromethane and stirred for 30 min. After that, 2 mL methanol solution was added and the mixture was stirred overnight under nitrogen protection at room temperature. The organic solvent was removed by evaporation, and the crude product was purified by recrystallization of acetonitrile and ether, with a yield of 65% (2.03 g, 6.5 mmol). ^{1}H NMR (500 MHz, MeOD-d_4) δ 7.99 (d, J = 8.7 Hz, 1H), 7.95 – 7.87 (m, 2H), 7.80 (d, J = 2.2 Hz, 1H), 7.60 – 7.49 (m, 2H), 7.38 (dd, J = 8.7, 2.2 Hz, 1H). ^{13}C NMR (126 MHz, MeOD-d_4) δ 158.01, 135.03, 133.60, 133.28, 131.03, 128.71, 127.95, 127.59, 124.67, 124.32, 54.65. HRMS (ESI): m/z $[M + H]^+$ calcd for $C_{11}H_{12}N_3$: 186.1031; found: 186.1030.

Supplementary Table 1 | The crystal parameters of NGAI (1-(naphthalen-2-yl) guanidinium iodide).

**Parameters**	
formula	$C_{11}H_{12}IN_3$
T (K)	100
color	colorless
crystal system	monoclinic
space group	$P2_1/c$
**a** (Å)	13.2842(6)
**b** (Å)	5.9878(2)
**c** (Å)	14.6297(6)
**α** (°)	90
**β** (°)	92.2727(15)
**γ** (°)	90
Volume (Å^3)	1162.78(8)
Z	4
ρ_{cal} (g cm^{-3})	1.789
Radiation	MoKα (λ = 0.71076)
F (000)	608.0
Goodness-of-fit on F^2	1.136
Final R indexes [I ≥ 2σ (I)]	R_1 = 0.0139, wR_2 = 0.0322
Final R indexes [all data]	R_1 = 0.0156, wR_2 = 0.0335

Computational spectral reconstruction.

Xenon light was monochrome by a monochromator, the exit slit is controlled to 1 mm, and the current-voltage curve of the device is scanned by Keithley 2400 source meter under different irradiance and wavelength. The irradiance of light at each monochromatic wavelength is measured using a standard silicon photodetector. The response matrix of the responsivity of the device under different irradiance with voltage and wavelength is calculated and used as the reference standard data. The responsivity is calculated by following Eq. (1):

$$R_{es} = \frac{J_{light} - J_{dark}}{I_0} \tag{1}$$

where R_{es} is responsivity, and J_{light} is the current density under light. J_{dark} is the current density under dark. I_0 is the power density of the light. The EQE of the photodetector can be calculated by the following Eq. (2):

$$\mathrm{EQE} = \frac{R_{es} h\nu}{q} \tag{2}$$

where EQE is external quantum efficiency. h is Planck constant (h = 6.626×10^{-34} J s). υ is the frequency of the light. q is the absolute value of electron charge (q = 1.602×10^{-19} C).

The current density and voltage curve of the photodetector under the unknown light is measured. The corresponding spectral curve was calculated by the algorithm reported by Yoon and Sun et al[2]. The difference is that we used reverse bias tunable response as the source in the learning process.

Supplementary Figures and Discussions

Synthesis and characterization of 1-(naphthalen-2-yl)guanidinium iodide.

The NGAI was synthesized in two steps, as illustrated in Supplementary Fig. 1a. Boc is the t-butyloxycarboryl group. DIPEA is a kind of base to improve the nucleophilicity of $–NH_2$. The chemicals of TMSI and MeOH are used to produce dried hydroiodic acid (HI) and remove the Boc group. Supplementary Fig. 1b is the ^{1}H NMR spectrum of NGAI. Supplementary Fig. 1c is the ^{13}C NMR spectrum of NGAI.

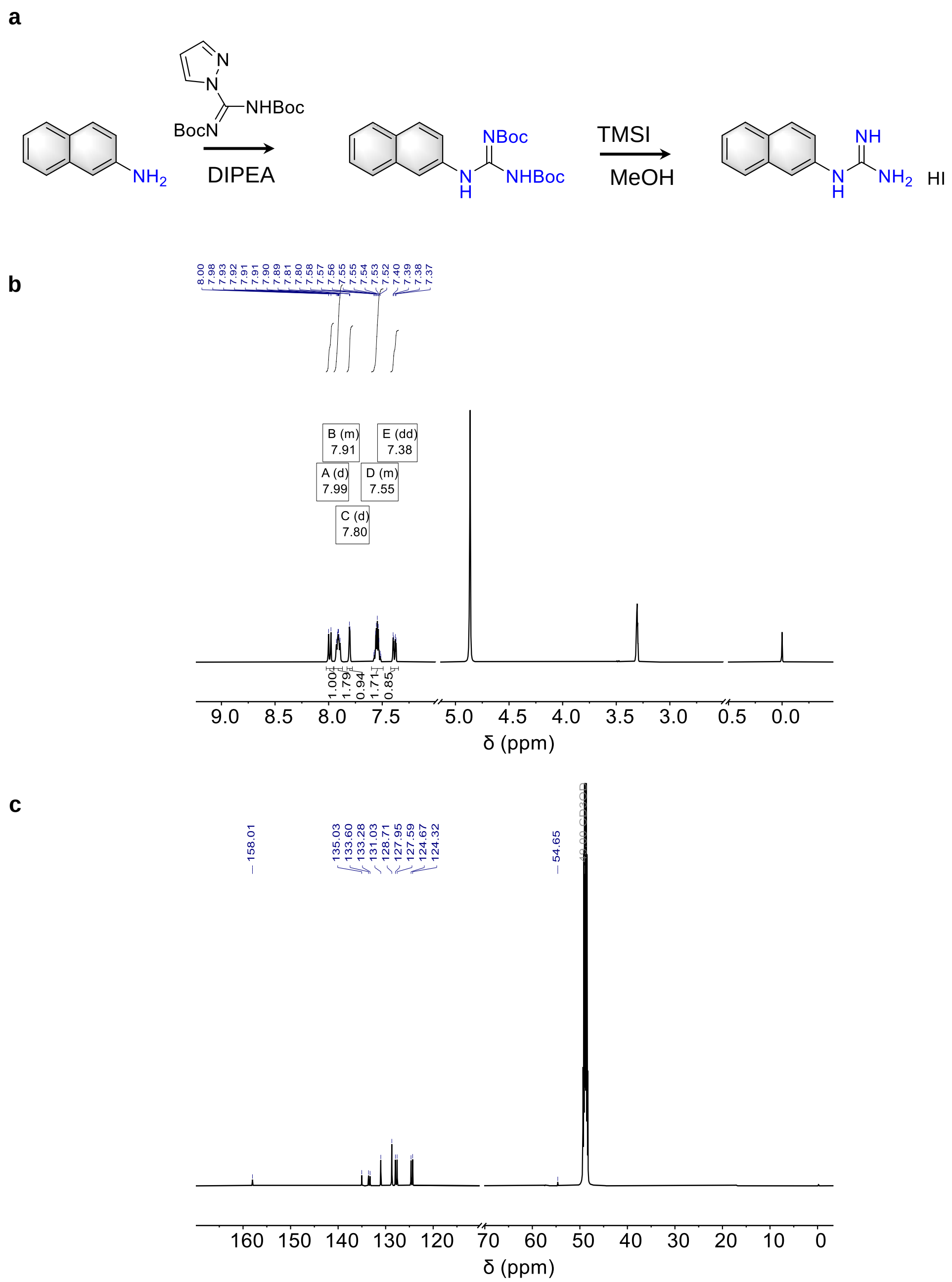

Supplementary Fig. 1 | ^{1}H NMR of 1-(naphthalen-2-yl) guanidinium iodide. ^{13}C NMR of 1-(naphthalen-2-yl) guanidinium iodide. a, The synthetic route of NGAI. **b,** ^{1}H NMR of NGAI. **c,** ^{13}C NMR of NGAI.

The crystal structure of NGAI exhibits a highly ordered layered arrangement, as illustrated in Supplementary Fig. 2. The crystal system of NGAI is monoclinic with the lattice parameters of **a** = 1.33 nm, **b** = 0.60 nm, **c** = 1.46 nm, **α** = 90°, **β** = 92.3°, **γ** = 90°. NGAI are naphthalenes derivatives with guanidinium groups where naphthyl groups are hydrophobic building blocks and guanidinium groups are hydrophilic groups. Iodide ions are chelated by guanidinium groups (Supplementary Fig. 2d).

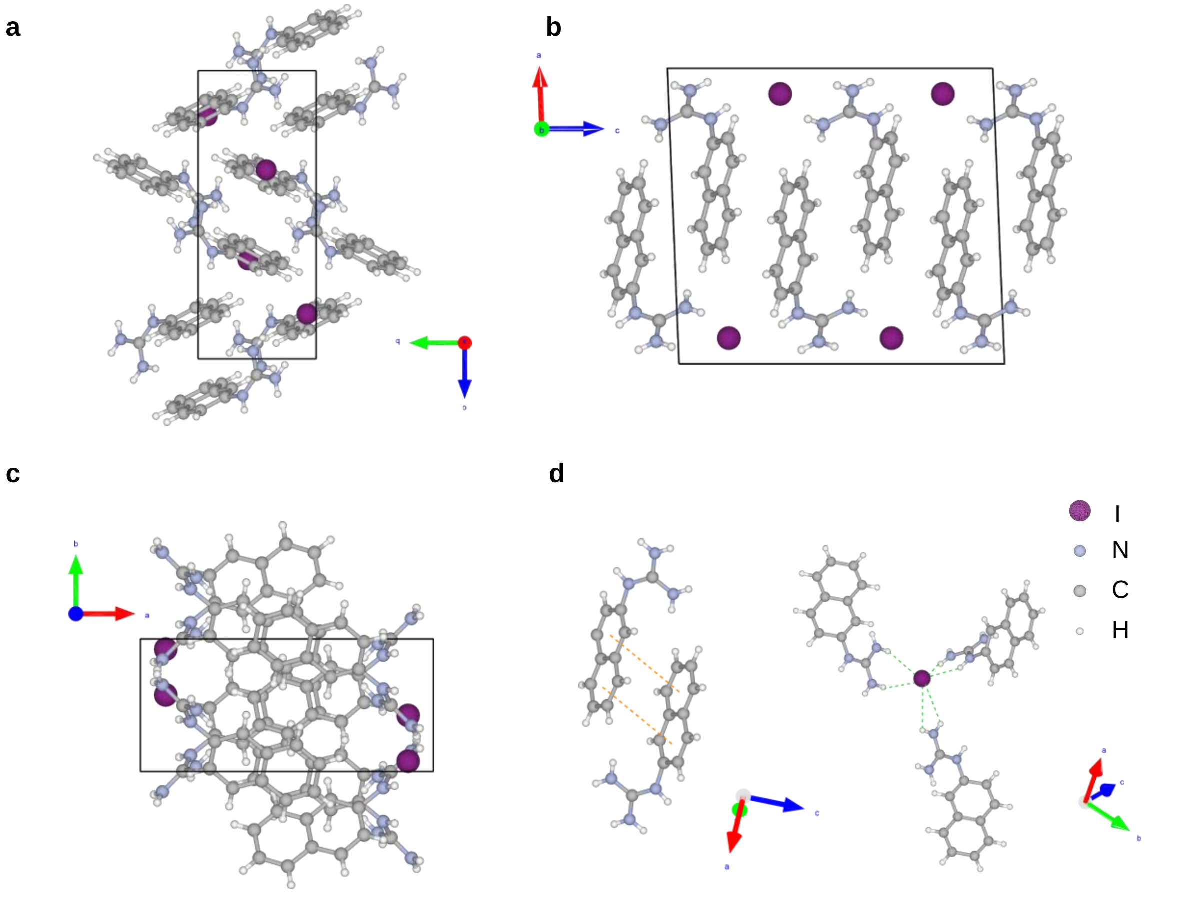

Supplementary Fig. 2 | The crystal structure of NGAI. a, The crystal structure of NGAI (perpendicular to axis a). **b,** The crystal structure of NGAI (perpendicular to axis b). **c,** The crystal

structure of NGAI (perpendicular to axis c). **d,** π-π interaction and hydrogen bond interaction in the crystal of NGAI.

The characterization of the 1-(naphthalen-2-yl)guanidinium iodide - Lead(II) iodide complex

The morphology of the NGAI and NGAI-PbI_2 complex in the polar solvent was investigated using TEM. Samples are prepared in the following way. The NGAI (3.1 mg) was dissolved in a mixed solvent of ACN: DMF: 2-Me = 3: 1: 1 (v/v, 1.5 mL), with or without PbI_2 (4.6 mg), to obtain the solutions of NGAI and NGAI-PbI_2. The solution was deposited onto Holey Carbon Grids (HCG) and subsequently air-dried. The TEM images of the NGAI-PbI_2 complex are depicted in Supplementary Fig. 3a and Supplementary Fig. 3b, showcasing its nanoparticle morphology. The crystallinity of NGAI-PbI_2 complex can be enhanced by appropriately reducing the amount of DMF in the mixed solvent. The amplified TEM image revealed the presence of the (102) plane in PbI_2 (Supplementary Fig. 3b). Supplementary Fig. 3c shows the (102) plane of the PbI_2 crystal. The average diameter of nanoparticles is ~4.7 nm. The radius of nanoparticles approaches the reported Bohr radius of perovskites[3]. Therefore, it is reasonable to propose that the NGAI-PbI_2 complex assumes the structure of the quantum dot. Subsequently, we fabricated films comprising NGAI, NGAI-PbI_2, and PbI_2 to obtain XRD spectra and absorbance spectra. The NGAI, NGAI (w 1 equiv. PbI_2), and PbI_2 were dissolved in the mixed solvent of ACN: DMF: 2-Me = 3: 1: 1 (v/v) to obtain the precursor of 0.2 M (PbI_2 can not be dissolved in the mixed solvent in this step. Thus,

the PbI_2 precursor was saturated solution in this condition.). Each kind of precursor was spray-coated onto the substrate to obtain the film. The XRD spectra are shown in Supplementary Fig. 3d (The peak of ~10 degrees is the system noise of the instrument.). The diffraction peak identification of NGAI is referred to as the single crystal data. Peaks of NGAI-PbI_2 mainly come from (200) and (112) of NGAI. No peaks corresponding to PbI_2 in the NGAI-PbI_2 complex sample were observed, and the peaks of NGAI-PbI_2 exhibit distinct difference compared to those of NGAI. Therefore, it is plausible that the interaction between NGAI and PbI_2 in the NGAI-PbI_2 film could induce a reorientation of NGAI molecules. The crystallinity of PbI_2 is simultaneously diminished by NGAI. Supplementary Fig. 3e shows the (100) plane of the NGAI crystal. The Supplementary Fig. 3f presents the absorbance spectra of NGAI, NGAI-PbI_2, and PbI_2. It is observed that the absorbance spectrum of NGAI-PbI_2 exhibits a blue shift compared to that of PbI_2, which can potentially be attributed to the quantum size effect.

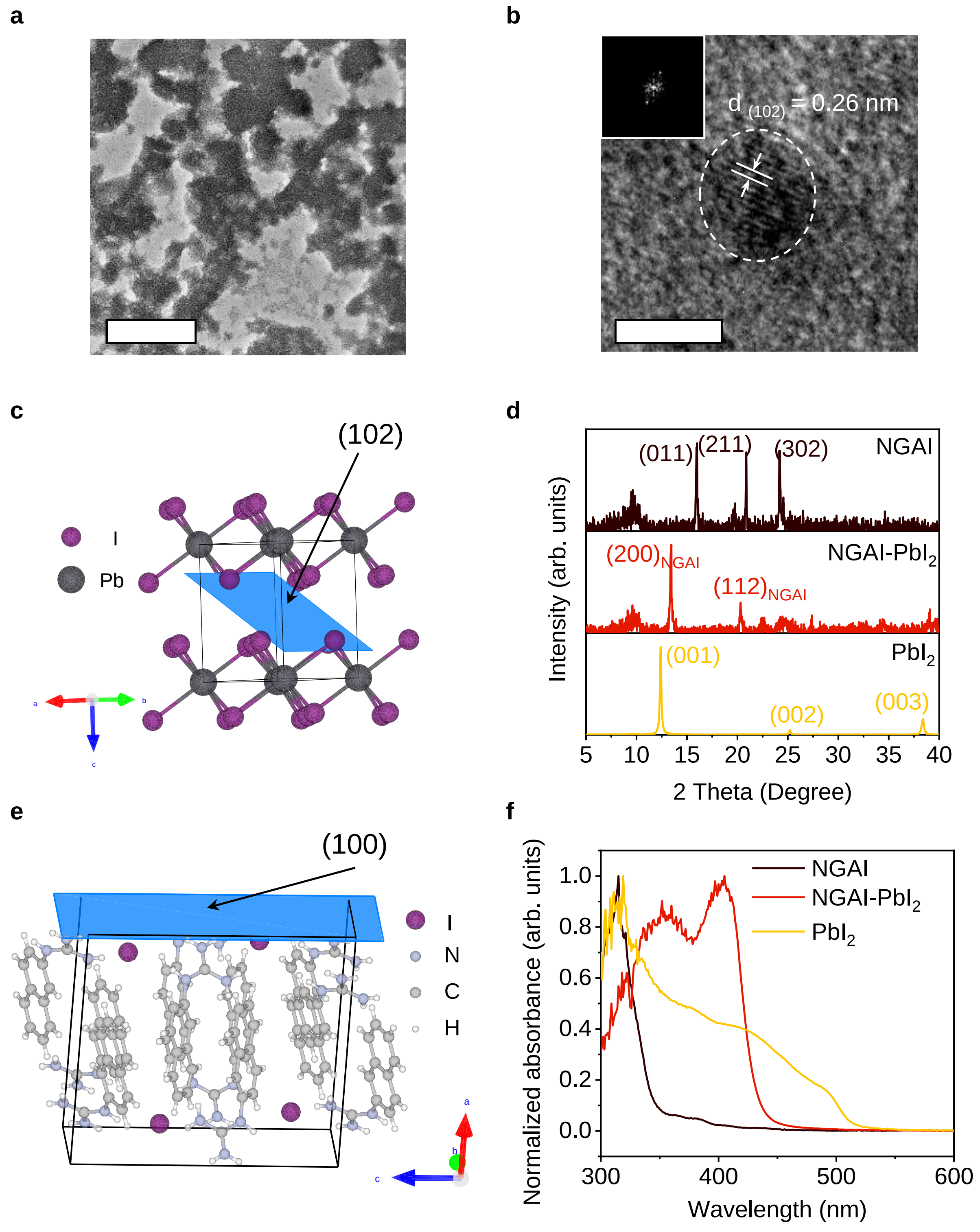

Supplementary Fig. 3 | The interaction between NGAI and PbI_2. a, The TEM image of the NGAI-PbI_2 complex, scale bar: 200 nm. **b,** The high-resolution transmission electron microscopy

(HRTEM) image of the NGAI-PbI_2 complex. Inset is the FFT image of the NGAI-PbI_2 complex, scale bar: 5 nm. **c,** The lattice plane (102) of the PbI_2 crystal. **d,** The XRD spectra of films (NGAI, NGAI-PbI_2, PbI_2) fabricated by the spray-coating method. **e,** The lattice plane (100) of the NGAI crystal. **f,** The absorbance spectra of films fabricated by spray-coating.

The characterization of perovskite (with 1-(naphthalen-2-yl)guanidinium iodide) film

The $FAPbI_3$ films were fabricated by spray coating with varying amounts of NGAI in order to investigate the impact of NGAI dosage. The absorbance spectra of $FAPbI_3$ films, fabricated by spray-coating with varying amounts of NGAI, are presented in Supplementary Fig. 4a. The increase in NGAI amounts resulted in a rise of the peak at ~380 nm. This peak may originate from the NGAI-PbI_2 complex, supported by its similar absorbance spectra but with a blue shift. The TEM image of $FAPbI_3$ (w 50%$_{mol}$ NGAI) is shown in Supplementary Fig. 4b. The TEM sample was prepared in the following process. The precursor solution (~1 mg mL^{-1} $FAPbI_3$ with 50%$_{mol}$ NGAI and 30%$_{mol}$ MACl) was deposited onto HCG for TEM analysis. The solvent used was a mixture of ACN, DMF, and 2-Me in a ratio of 3: 1: 1 (v/v). Subsequently, the HCG sample underwent annealing at 120℃ for a duration of 15 minutes. The morphology of $FAPbI_3$ (with 50%$_{mol}$ NGAI) also exhibits a nanoparticle structure. The average diameter of the nanoparticles is ~3.9 nm, which is smaller compared to the nanoparticles formed by the NGAI-PbI_2 complex (Supplementary Fig. 3a). These differences should come from the interaction between FA^+/MA^+

and $[PbI_6]^{4-}$. The transient absorption spectra are shown in Supplementary Fig. 4c and Supplementary Fig. 4d. No discernible peak corresponding to low dimensional perovskite was observed in the spray-coated film of $FAPbI_3$ perovskite (w30%$_{mol}$ NGAI). The negative peak at ~500 nm was attributed to PbI_2. The presence of NGAI induced a transformation in the crystallization process of the $FAPbI_3$ film through the formation of the NGAI-PbI_2 complex. Because of the highly ordered molecular arrangement of NGAI with rigid structures, the crystallization process is also templated by NGAI. The addition of NGAI promotes the preferential formation of crystalline grains exposed to the (111) lattice plane, as observed in Fig. 1h, in the polycrystalline films. The SEM images of $FAPbI_3$ films with varying concentrations (0%$_{mol}$ ~ 30%$_{mol}$) fabricated through spray-coating are presented in Supplementary Fig. 4e. With the amount of NGAI increased grain boundary of the $FAPbI_3$ film broadened. The films tend to form bigger grains with the addition of a small amount of NGAI. Excess amounts of NGAI inhibit the growth of $FAPbI_3$. We considered that the NGAI mainly exists in the grain boundary.

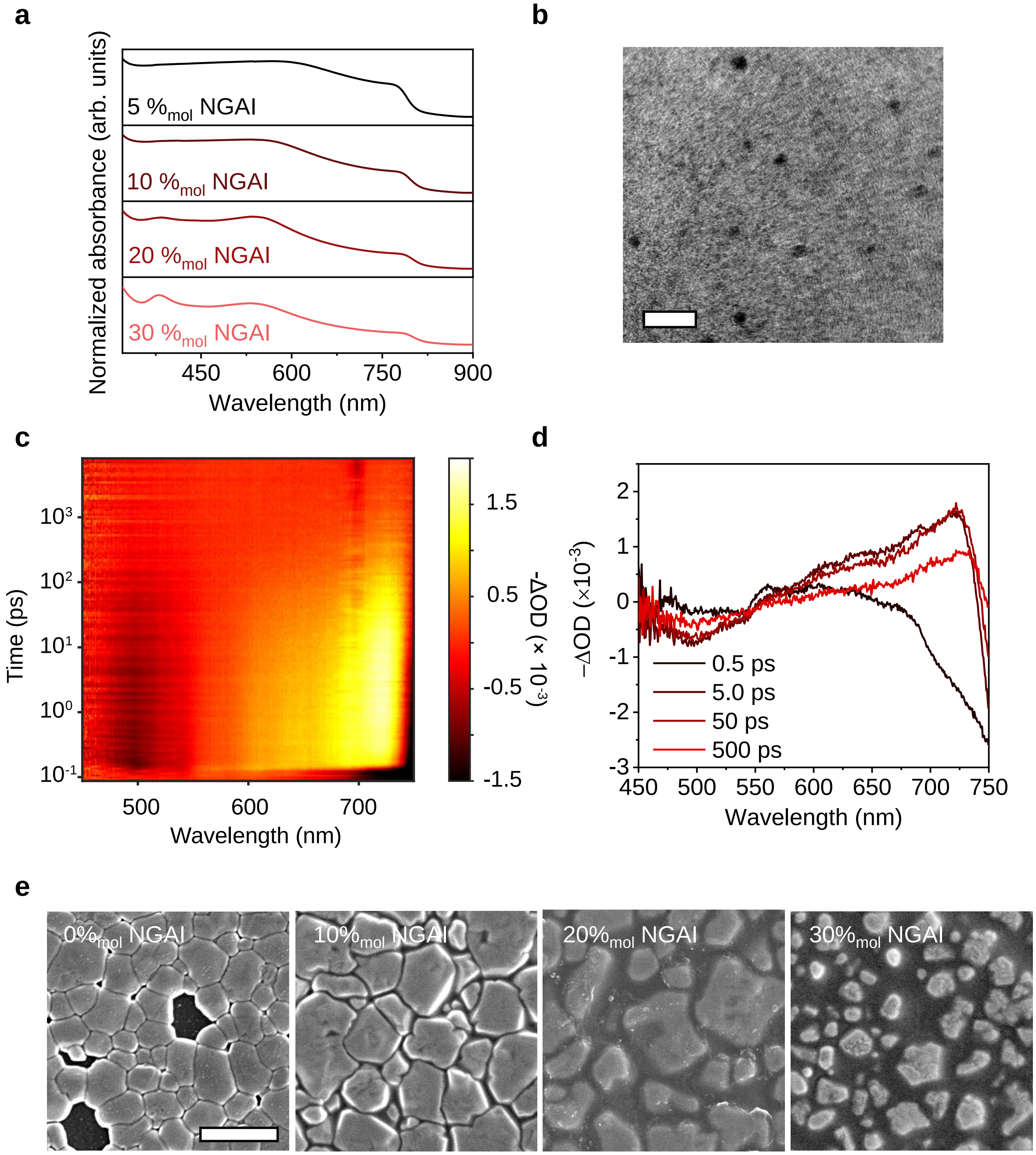

Supplementary Fig. 4 | The interaction between NGAI and $FAPbI_3$. a, The absorbance spectra of $FAPbI_3$ (w 5%$_{mol}$ ~ 30%$_{mol}$ NGAI) films fabricated by spray-coating. **b,** The TEM image of $FAPbI_3$ (w 50%$_{mol}$ NGAI), scale bar: 20 nm. **c, d,** The transient absorption spectrum of the $FAPbI_3$

(w 30%$_{mol}$ NGAI) film. **e,** The SEM images of $FAPbI_3$ (0%$_{mol}$ ~ 30%$_{mol}$ NGAI) films fabricated by spray-coating method, scale bar: 2 μm.

To study the interaction between the NGAI and PbI_2 in the solution state, ^{1}H NMR spectra of NGAI, NGAI (w 1 equiv. PbI_2), and NGAI (w 1 equiv. $FAPbI_3$) were obtained (Supplementary Fig. 5a). The concentration of NGAI is 0.02M. The solvent is ACN (CD_3CN-d_3) with adding 1% 2-Me. In the case of NGAI (w 1 equiv. $FAPbI_3$), 0.3 equiv. MACl was added to better simulate the precursor. After adding PbI_2, chemical shifts of NGAI exhibit a down-field shift. The biggest change is the peak of H (1, 7) and H (2, 3). These changes reflect the electron cloud differences caused by the interaction between PbI_2 and guanidinium because H (1, 7) and H (2, 3) are close to the guanidinium in space. After adding FAI, chemical shifts (H (1) and H (2, 3)) of NGAI exhibit a slightly up field shift, which is possibly influent by the interaction between FAI and PbI_2.

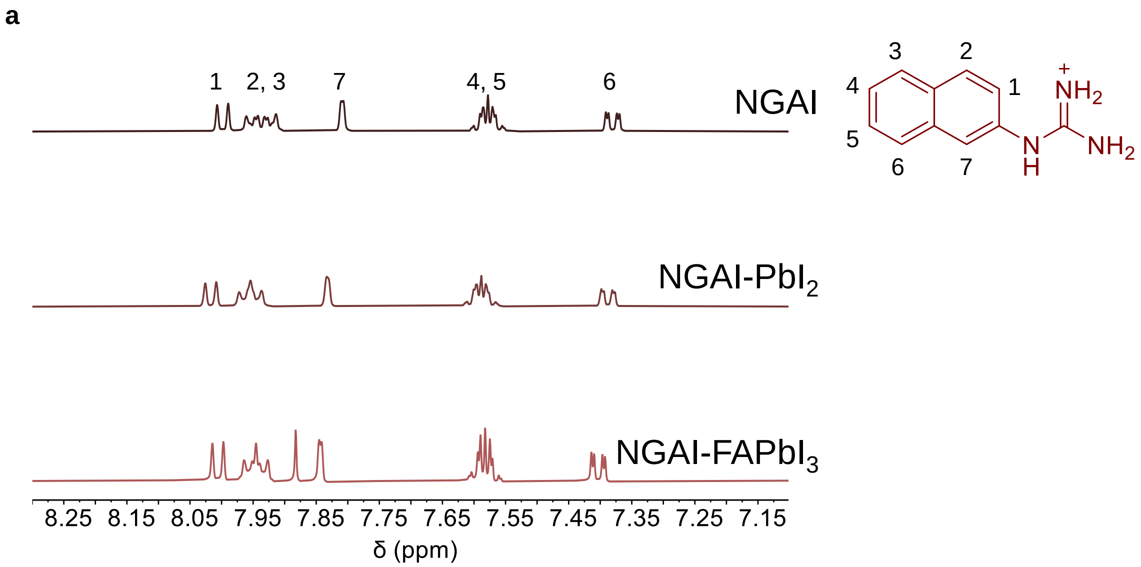

Supplementary Fig. 5 | The interaction in the solution state. a, The ^{1}HNMR spectra of NGAI, $NGAI\text{-}PbI_2$ complex and $NGAI\text{-}FAPbI_3$ complex.

NGAI exhibits lower solubility (0.032 mg mL^{-1}) in water, which is lower than other hydrophobic ammonium salt widely used (Supplementary Fig. 6a). The NGAI augments the perovskite's chemical inertness. NGAI are distributed in the grain boundary inhibiting the invasion of water and oxygen. Additionally, facet engineering can also enhance the stability of perovskite films[4]. The stability of $FAPbI_3$ (w $10\%_{mol}$ NGAI) film was monitored by absorbance spectra. The film was aged for 30 days without any decomposing at R.H. 40% ~ 85% (Supplementary Fig. 6b). We conducted stability tests on the photodetector under continuous illumination conditions (700 nm, -0.6 V) at R.H. 40% ~ 85%. The detector, when unencapsulated, demonstrated the capability to maintain an initial responsivity exceeding 90% for a period exceeding 30 days (Supplementary

Fig. 6c). Additionally, we presented the photocurrent-voltage (*J* - *V*) characteristics of the photodetector after 30 (Supplementary Fig. 6d).

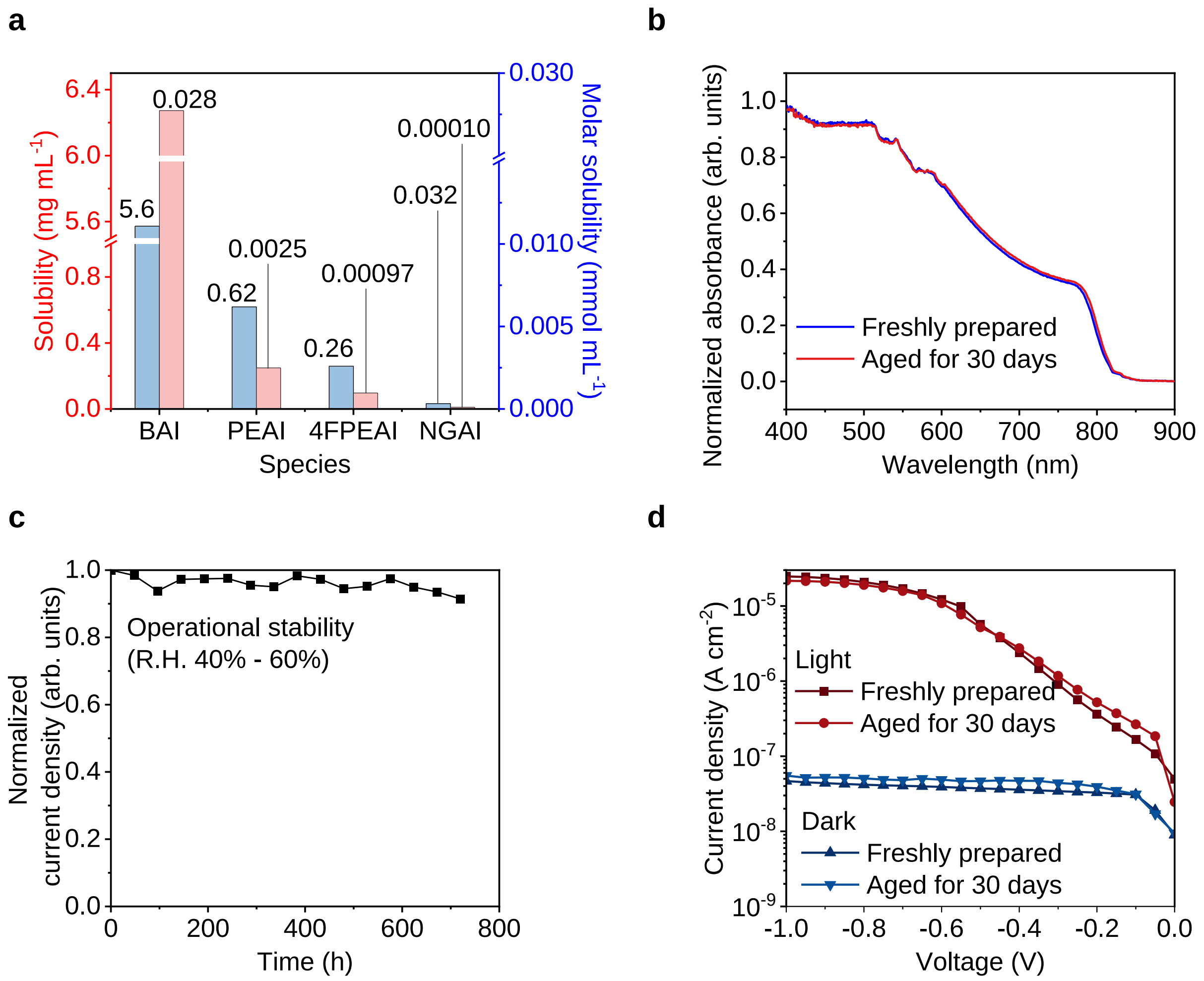

Supplementary Fig. 6 | The stability of the $FAPbI_3$ (w NGAI) film fabricated by spray-coating. a, The solubility of different hydrophobic ammonium salt. BAI is butylamine hydroiodide. PEAI is 2-phenylethanamine hydroiodide. 4FPEAI is 2-(4-fluorophenyl) ethan-1-amine hydroiodide. **b,** The absorbance of the $FAPbI_3$ (w $10\%_{mol}$ NGAI) freshly prepared and after aging for 30 days at ambient (R.H. 40% ~ 85%). **c,** The operational stability of the photodetector (R.H. 40% ~ 85%). **d,** The *J* - *V* curve of the photodetector freshly prepared and aged for 30 days.

The photodetector performance.

The device structure of the perovskite photodetector fabricated by spray-coating is Cr/PTAA/$FAPbI_3$(w 10%$_{mol}$ NGAI)/C_{60}/BCP/Cr/Au. The Au side serves as the incident side for light illumination. The top metal electrode (Cr and Au) is designed to have a thin thickness in order to maintain high transmittance[5]. Supplementary Fig. 7a shows the dark current density and the light current density of the photodetector. The dark current density of the photodetector is 2.20×10^{-8} A cm^{-2} at -1.0 V bias. Lower dark current density benefits from the improved density of the film by adding NGAI. The light current density of the photodetector at 532 nm, 10 μW cm^{-2}, and -1.0 V bias is 5.1×10^{-5} A cm^{-2}. The responsivity of the photodetector is 5.1 A W^{-1} and calculated by Eq. (1). The EQE of the photodetector is 1180%. The EQE higher than 100% results from the external injection of carriers by the power source. Supplementary Fig. 7b shows the trap density of state (tDos) of the photodetectors fabricated by spray-coating ($FAPbI_3$ w 10%$_{mol}$ NGAI) and spin-coating ($FAPbI_3$ w/o NGAI). The tDos (~10^{-15} ~ 10^{-17} eV cm^{-3}) of photodetectors fabricated by spray-coating ($FAPbI_3$ w 10%NGAI) is higher than that (~10^{-11} - 10^{-15} eV cm^{-3}) of photodetectors fabricated by spin-coating ($FAPbI_3$ w/o NGAI). The tDos was evaluated by measuring the frequency and capacitance of the device. The demarcation energy ($E\omega$) with the applied frequency by the following Eq. (3)[6].

$$E_{\omega} = k_B T \ln\left(\frac{\omega_0}{\omega}\right) \tag{3}$$

where ω is the applied angular frequency. The ω_0 we used is the published data[7]. k_B is the Boltzmann constant, and T is the absolute temperature. The distribution of the tDos can be calculated by Eq. (4).

$$tDos(E_\omega) = -\frac{V_{bi}}{qW}\frac{dC}{d\omega}\frac{\omega}{k_B T} \tag{4}$$

where V_{bi} is the built-in potential. W is the width of the depletion region, and the thickness of the active layer is employed as W. q is the elementary charge. Meanwhile, the fabrication of photodetectors by spray-coating ($FAPbI_3$ without NGAI) poses significant challenges. The resulting photodetector exhibits substantial leakage current and fails to function effectively.

Devices of ITO/PTAA/$FAPbI_3$ (w 10%$_{mol}$ NGAI)/PTAA/Cr (Device 1) and ITO/C_{60}/$FAPbI_3$ (w 10%$_{mol}$ NGAI)/C_{60}/Cr (Device 2) were prepared to analyze the carrier transport behavior of the $FAPbI_3$ (w 10%$_{mol}$ NGAI) film fabricated by spray-coating process (Supplementary Fig. 7c). Voltages of the trap filling limit (V_{TFL}) of Device 1 and Device 2 are 1.19 V and 2.59 V. The average thickness of films is ~2.00×10^{-4} cm measured by the step profiler. The dielectric constant ($\varepsilon_0\varepsilon$) is 1.63×10^{-11} F cm^{-1}. The trap density (N_t) of films can be calculated in the following Eq. (5)

$$N_t = \frac{2\varepsilon_0\varepsilon V_{TFL}}{qL^2} \tag{5}$$

where L is the thickness of the film. The hole N_t of the $FAPbI_3$ (w 10%$_{mol}$ NGAI) film is 6.03×10^{15} and the electron N_t of the $FAPbI_3$ (w 10%$_{mol}$ NGAI) film is 1.30×10^{16}. The mobility of $FAPbI_3$ (w 10%$_{mol}$ NGAI) film can also be analyzed by Mott–Gurney law (Eq. (6)).

$$J = \frac{8}{9}\varepsilon_0\varepsilon\mu\frac{(V-V_{bi})^2}{L^3} \tag{6}$$

where V_{bi} is built-in potential. μ is the mobility. J is the current density. The hole moblity(μ_h) of the $FAPbI_3$ (w $10\%_{mol}$ NGAI) film 6.76×10^{-4} cm^2 V^{-1} s^{-1}. The electron moblity(μ_e) of the $FAPbI_3$ (w $10\%_{mol}$ NGAI) film 3.18×10^{-4} cm^2 V^{-1} s^{-1}.

According to the carrier transport behavior and the trap density states of the $FAPbI_3$ (w $10\%_{mol}$ NGAI) film, the gain of the photodetector is induced by the traps of electrons at the surface of perovskite after adding NGAI, which leads to the hole injection (Supplementary Fig. 7d). The photodetector also performs the noise current of ~10^{-13} A $Hz^{-0.5}$ and shown in the Supplementary Fig. 7e. The detectivity (D^*) of photodetector is ~10^{13} Jones (Supplementary Fig. 7f) at -1.0 bias, which is calculated by the following Eq. (7).

$$D^* = \frac{\sqrt{AB}R_{es}}{i_{noise}} \tag{7}$$

where A is the effective area of the photodetector. B is the bandwidth.

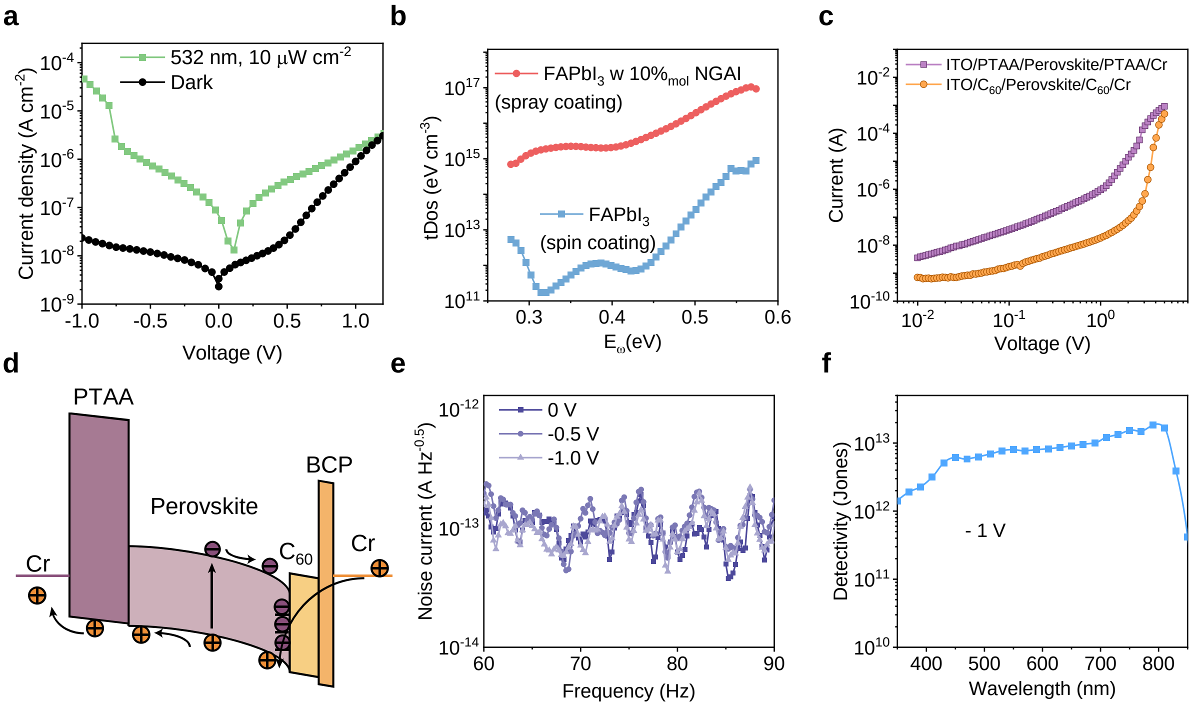

Supplementary Fig. 7 | The performance of the photodetector. a, The current density (*J*) – voltage (*V*) curve of the photodetector under light (532 nm, 10 μW cm^{-2}) and dark. **b,** The tDos of photodetectors fabricated by spray-coating ($FAPbI_3$ w $10\%_{mol}$ NGAI) and spin-coating ($FAPbI_3$ w/o NGAI). **c,** The space charge limited current (SCLC) of the film fabricated by spray-coating ($FAPbI_3$ w $10\%_{mol}$ NGAI). **d,** The schematic diagram of the energy level structure of the photodetector. The perovskite is $FAPbI_3$ (w $10\%_{mol}$ NGAI). **e,** The noise current of the $FAPbI_3$ ($10\%_{mol}$ NGAI) photodetector at different biases. **f,** The detectivity of the $FAPbI_3$ ($10\%_{mol}$ NGAI) photodetector at the bias of -1.0 V.

Reconstructing the spectra of two adjacent wavelengths ranging from 590 nm to 610 nm allowed us to evaluate the resolution of the computational spectrometer. The photodetector exhibits the ability to discriminate between wavelengths of 597.5 nm and 602.3 nm, with a resolution of ~4.7 nm (Supplementary Fig. 8a). During the learning process, each step of wavelength is 2 nm. The FWHM of the incident light is ~2 nm, which is calculated by the reciprocal linear dispersion of the instrument. The reference spectra of monochromatic light are also calculated by reciprocal linear dispersion in the following Eq. (8).

$$\frac{\Delta\lambda}{\Delta x_s} = \frac{d_g \cos\psi}{nF} \quad (8)$$

where $\frac{\Delta\lambda}{\Delta x_s}$ is the reciprocal linear dispersion. d_g, ψ and F are the spacing of grating grooves, diffraction angle, and effective focal length of the system, respectively, and n is the diffraction order.

The responsivity of the photodetector is changed by the irradiance of the incident light[2]. The variation in incident light depth is attributed to the differences in irradiance levels. Therefore, the reconstruction of spectra from arbitrary combinations of light intensity and wavelength can be achieved by establishing a model that relates the light intensity to the voltage-wavelength-responsivity matrix. The responsivities of photodetectors at different voltages and wavelengths are presented in Supplementary Fig. 8c - 8e, where the incident light irradiance is ~10^1 and ~10^{-1} μW cm^{-2}, as referenced in Supplementary Fig. 8b.

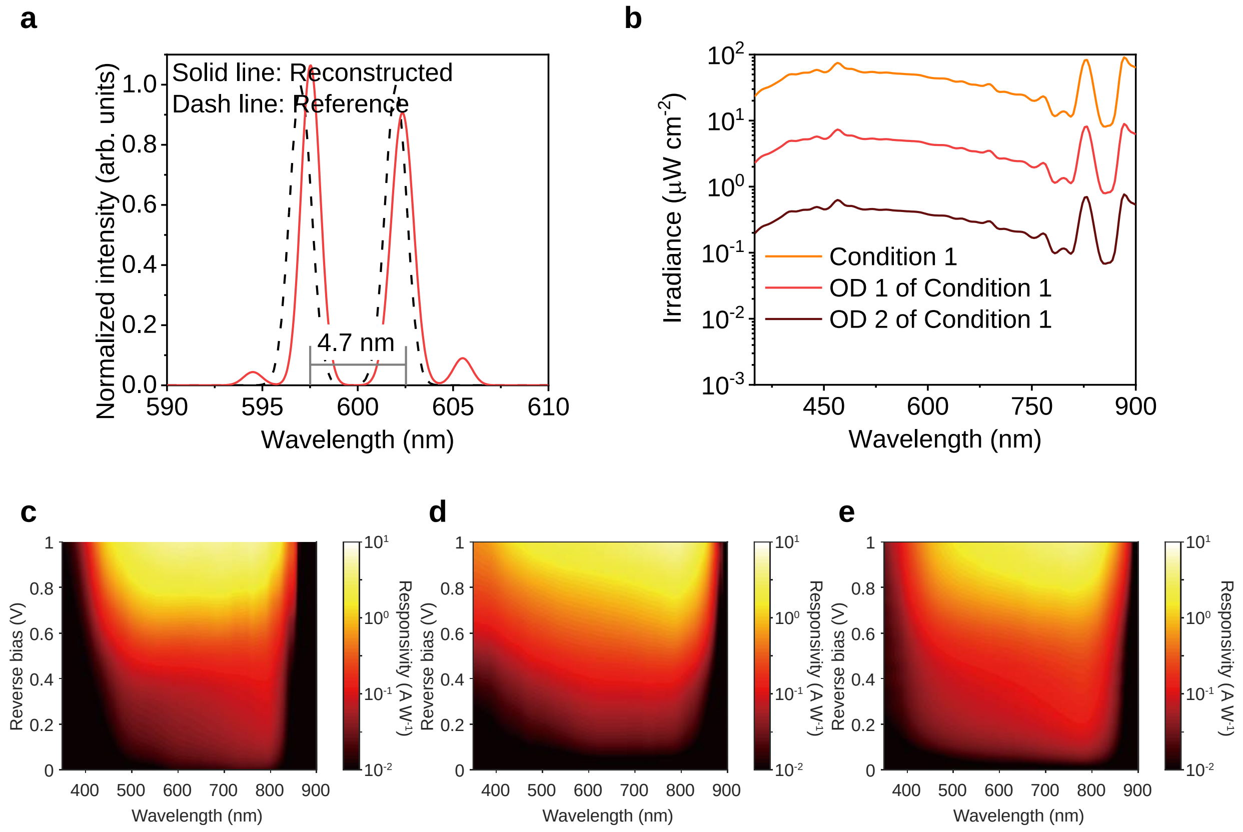

Supplementary Fig. 8 | The performance of the computational spectrometer. a, The spectra to evaluate the resolution of the computational spectrometer. Solid lines are the spectra

reconstructed from the computational spectrometer. Dash lines are obtained from the monochromator. **b,** The irradiance of the incident light. Condition 1 is the condition of Fig. 2a, 2b and Supplementary Fig. 8c. OD 1 of Condition 1 is the condition of the Supplementary Fig. 8d. Condition 3 is the condition of the Supplementary Fig. 8e. **c,** The responsivity of the photodetector at different voltages and wavelengths where the irradiance of incident light is same as Condition 1 in Supplementary Fig. 8b. **d,** The responsivity of the photodetector at different voltages and wavelengths where the irradiance of incident light is same as OD 1 of Condition 1 in Supplementary Fig. 8b. **e,** The responsivity of the photodetector at different voltages and wavelengths where the irradiance of incident light is same as OD 2 of Condition 1 in Supplementary Fig. 8b.

The single-pixel Fast Fourier Transform imaging and the color classification

An image can be regarded as a weighted sum of harmonic two-dimensional structured light patterns with different frequencies. The reconstruction of the image is possible if the weight corresponding to each frequency is known. In the imaging process, measurements involve obtaining the corresponding Fourier coefficients (weights) by known illuminations under specific illumination intensities. In theory, a two-dimensional image can be transformed into the spectral domain with the same number of pixels (complex Fourier coefficients) through the Fourier

spectrum transformation. A two-dimensional sinusoidal function (P (x, y; f_x, f_y)) can be expressed in terms of its spatial frequency (f_x, f_y) and initial phase (p).

$$P\left(x, y; f_x, f_y\right) = a + b \cdot \cos(2\pi f_x x + 2\pi f_y y + p) \tag{9}$$

where (x, y) represents the 2D Cartesian coordinates in the scene, a is equal to the average intensity of the image, b represents the contrast. Supplementary Fig. 9b shows the 2D typical four-step phase-shifting sinusoidal patterns (m = 47, 48, 49, 50, 51, 52, 53, 54). The Fourier coefficients are computed by measuring the intensity of light reflected from an object or image using a detector. Within the four-step phase-shifting method, the Fourier coefficients (C (f_x, f_y)) correspond to the initial phase (i. e. p = 0, π/2, π, π3/2) of the two-dimensional structured light emitted by the light source and can be expressed as Eq. (10)

$$C\left(f_x, f_y\right) = \frac{1}{2bk}\left\{\left[D_0\left(f_x, f_y\right) - D_\pi\left(f_x, f_y\right)\right] + j \cdot \left[D_{\pi/2}\left(f_x, f_y\right) - D_{3\pi/2}\left(f_x, f_y\right)\right]\right\} \tag{10}$$

where k is a scale factor whose value depends on the performance of the detector, D_p is response of the detector, j denotes the imaginary unit.

The reconstructed image Im (x, y) can be presented by following equation (Eq. (11)).

$$Im\,(x, y) = \int_{-\infty}^{+\infty}\int_{-\infty}^{+\infty}[D_0 - D_\pi] + j \cdot \left[D_{\pi/2} - D_{3\pi/2}\right] e^{j2\pi(f_x x + f_y y)} df_x df_y \qquad (11).$$

The reconstructed raw data from the single point imaging at different biases is presented in Supplementary Fig. 9c. The grayscale intensity of the images has been normalized to a consistent scale, enabling clear visualization of the variations in imaging at different biases. The noise can be reduced through background subtraction, smoothing, and linear weighting operation.

Supplementary Fig. 9d shows the photograph of the object. The signal of the photodetector is from the reflection of the front side of the Rubik's cube.

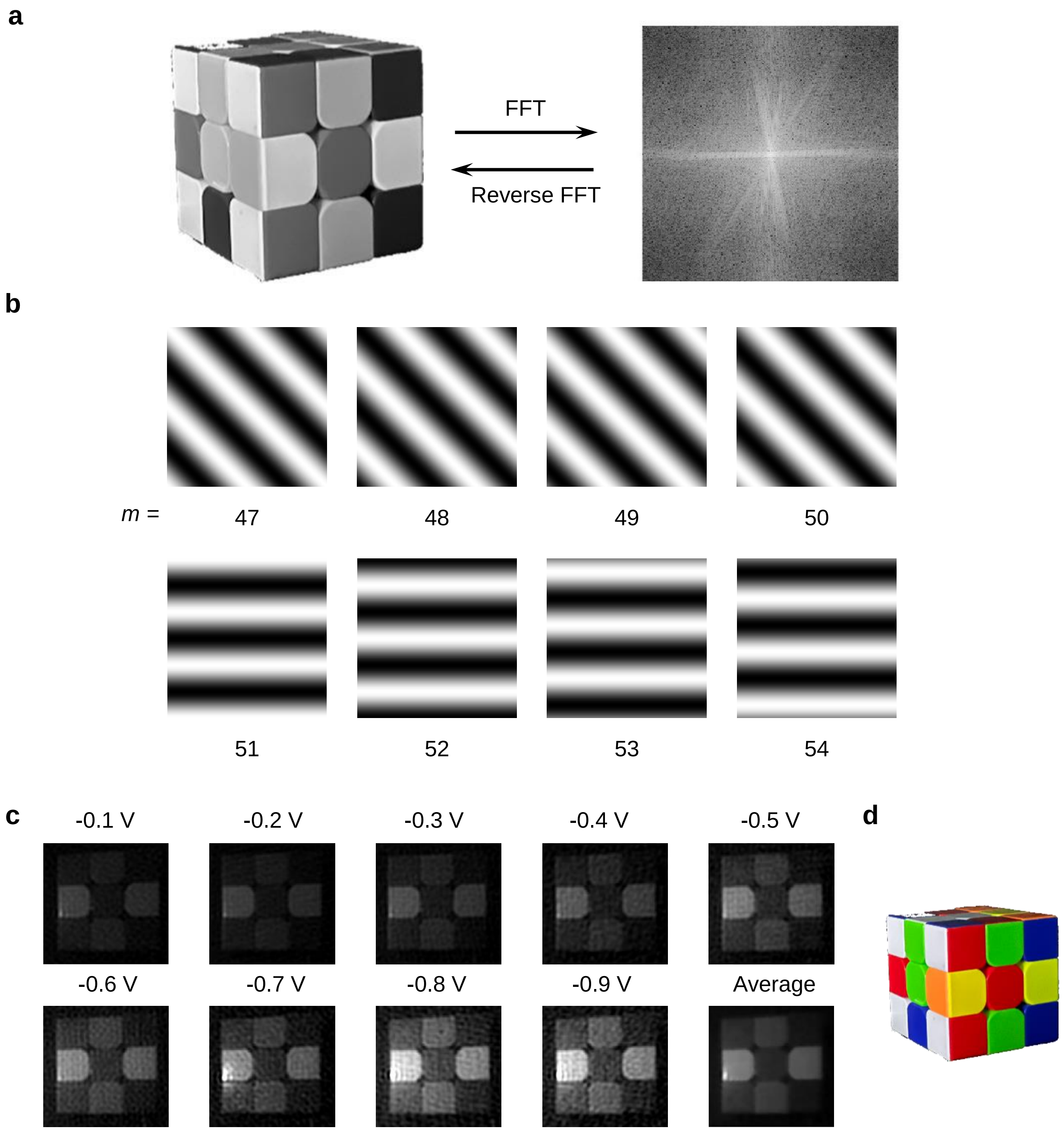

Supplementary Fig. 9 | The original imaging of the Rubik's cube by the single pixel imaging and the optical photograph of the Rubik's cube. a, The image is Fourier transformed before and

after **b,** The 2D patterns generated by projector when m = 47, 48, 49, 50, 51, 52, 53, 54. **c,** The original imaging of the Rubik's cube by the single pixel imaging under different biases obtained from the single pixel hemispherical photodetector. **d,** Photograph of the Rubik's cube.

Supplementary Fig. 10a is the confusion matrix generated from the training process. Each sample has 9 elements from V_1 to V_9 as the eigenvector. The main algorithm of the color classification is the K-nearest neighbor (KNN). The confusion matrix plot is to understand how the currently selected classifier performed in each class. For each color, we select 121 pixels (11 × 11) as the training group. The size of the image after cutting the background is 146 × 151. The accuracy of the prediction in the select group of the machine learning model is 100%. The ROC curve shows the true positive rate (TPR) versus the false positive rate (FPR) for different thresholds of classification scores, computed by the currently selected classifier. Supplementary Fig. 10b shows the ROC curve of the "Red" class.

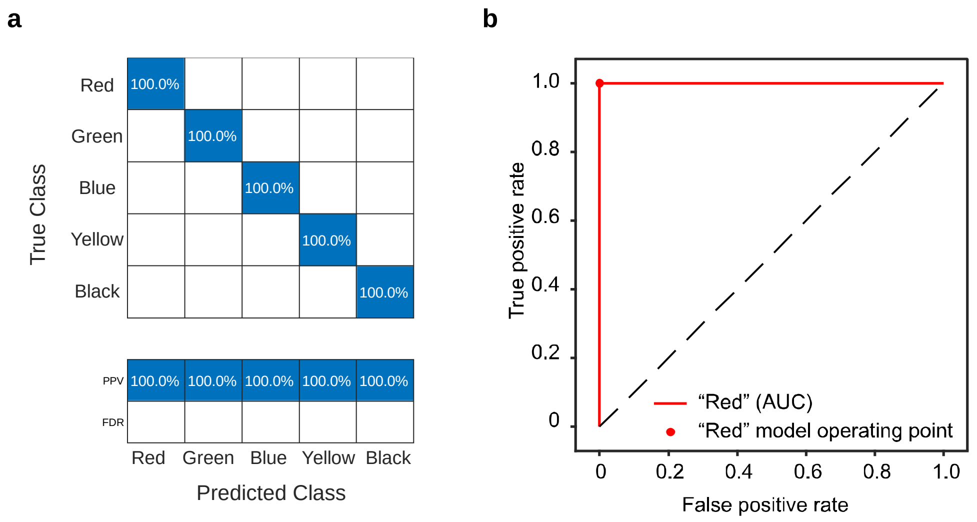

Supplementary Fig. 10 | The validation result of the color classification by KNN. a, The confusion matrix generated from the training process during KNN-assisted color classification. **b,** The ROC curve of the model after training.

To reduce the influence of uneven reflected light intensity on color classification, we conducted the following validation experiments. Initially, we measured the reflectance spectra of different facets of the Rubik's Cube under white LED illumination (Supplementary Fig. 11a). The spectra were optimized using Gaussian distribution curves (Supplementary Fig. 11b), and the corresponding photocurrent values were measured using a Si detector. The magnitude of the signal from the Si detector (S) can be expressed by the following equation (Eq. (12)):

$$S = \int_{w_1}^{w_2} R_{es} i dw \tag{12}$$

where w represents wavelength, w_1 and w_2 denote the lower and upper wavelength limits, respectively, R_{es} corresponds to the detector's responsivity at a specific wavelength (Supplementary Fig. 11c), and i signifies the intensity of the reflected light at a particular wavelength. This approach allows for the calibration of the relative intensity of light. Subsequently, the data previously categorized in the manuscript is multiplied by a calibration factor to approximate equalizing the light intensity. Following this step, the data is re-modeled and re-categorized, resulting in a validation of color re-classification effects (Supplementary Fig. 11d). Supplementary Fig. 11e shows the result after calibration and re-classification. Therefore, we approach with optimism the reconstruction of images using this differential method, despite the challenges posed by uneven light intensity that currently hinder the attainment of full-spectrum

imaging. Nevertheless, this approach presents a promising avenue for research. In subsequent investigations, refinement in color imaging is anticipated through algorithmic optimization and enhanced information capture.

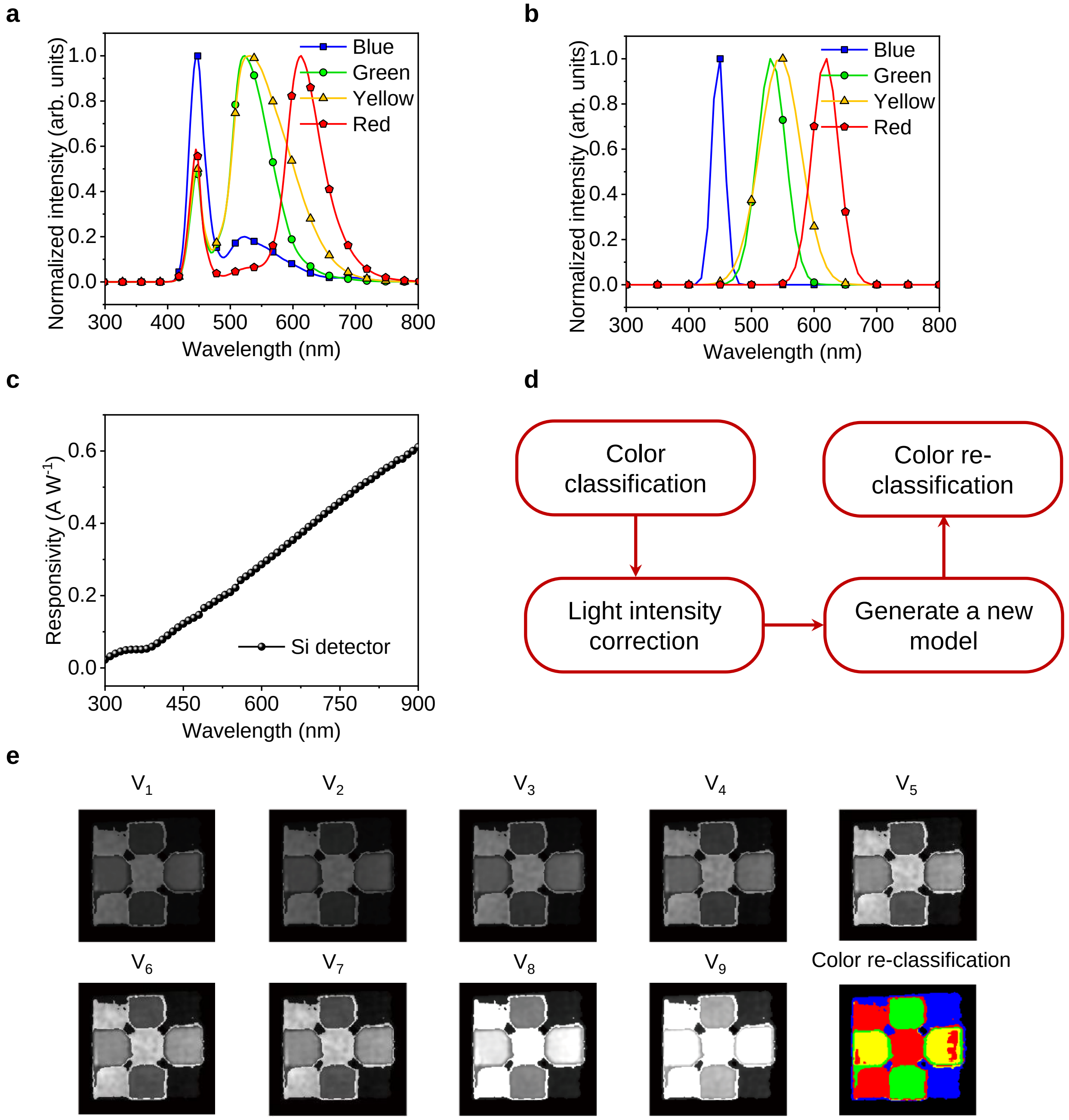

Supplementary Fig. 11 | The light intensity calibration and color classification revalidation. a, Reflectance spectra from each face of the Rubik's cube (Background is the spectrum of the white color LED). **b,** Reflectance spectra from each face of the Rubik's cube after optimizing. **c,** The responsivity of the Si photodetector. **d,** The process of the light intensity calibration and color classification revalidation. **e,** The images after light intensity calibration and the image after color re-classification.

The differential hemispherical photodetector for trace reconstruction

Supplementary Fig. 12 shows the analysis of effective incident flux intensity. The schematic diagram in Supplementary Fig. 12a illustrates the analysis of the effective incident flux intensity of a hemisphere under vertically incident light. Supplementary Fig. 12b presents a schematic diagram for analyzing the effective incident flux intensity of a hemisphere under incident light at an arbitrary position. Additionally, Supplementary Fig. 12c depicts a schematic diagram to analyze the effective incident flux intensity of a disc under incident light at an arbitrary position. Supplementary Fig. 12d is the schematic diagram to analyze the total effective incident flux intensity (φ) of the long pixel and short pixel. The model was simplified into two dimensions where $d\boldsymbol{S}$ can be simplified into $d\boldsymbol{l}_{\mathrm{c}}$. $\boldsymbol{l}_{\mathrm{c}}$ is the arc length under the light. The total effective incident flux intensity (φ) can be obtained by integral the $d\varphi$ on the arc and is shown in Eq. (13).

$$\int d\varphi = \int \mathbf{I} \cdot d\boldsymbol{l_c} = \int Ir \cos\beta_1 \, d\theta = \int \frac{rI_0}{4\pi R^3} |x\cos\theta - r| d\theta \tag{13}$$

The range of integration was classified for discussion and shown in Supplementary Fig. 12d. In the condition of long pixel, the range of integration can be divided into two segments (0 to α, 0 to $\frac{\pi}{2} - \alpha$) where α is the angle between the line through the light source and the bottom center of the hemisphere and the line through the foot point of the light source and the bottom center of the hemisphere. In the condition of the short pixel, the range of integration needs to be classified for discussion. If $\alpha < \frac{\pi}{4}$, the range of integration can be divided into two segments (0 to α, 0 to $\frac{\pi}{4} - \alpha$), similar to the long pixel. If $\alpha \geq \frac{\pi}{4}$, the range of integration is from $\alpha - \frac{\pi}{4}$ to α. The total effective incident flux of the arcs with the parameters (h and d) changing is normalized into the same scale and shown in Supplementary Fig. 12e and Supplementary Fig. 12f. The variation tendency of the long pixel and the short pixel is different. Thus, the design of different shapes of pixels can improve the accuracy of location tracking in theory.

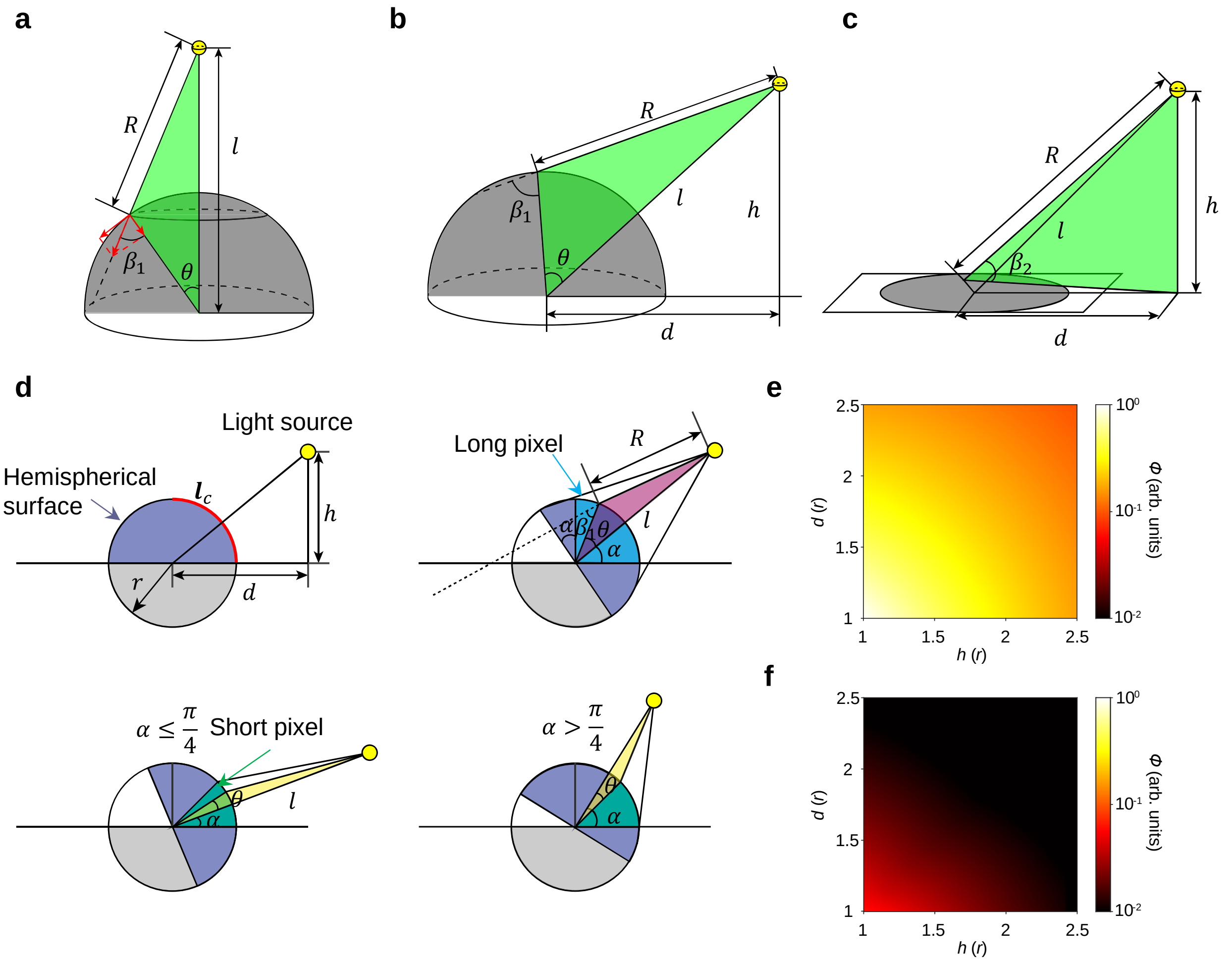

Supplementary Fig. 12 | The analysis of effective incident flux intensity of different pixels. a, The schematic diagram to analyze the effective incident flux distribution (vertical incident light). **b,** The schematic diagram to analyze the effective incident flux distribution (arbitrary position incident light). **c,** The schematic diagram to analyze the effective incident flux distribution (on the planar surface). **d,** The schematic diagram to analyze the effective incident flux intensity in different conditions. **e,** The total effective incident flux (φ) of 1/4 circle with different horizontal distance (d) and height (h). **f,** The total effective incident flux (φ) of 1/8 circle with different horizontal distance (d) and height (h).

The substrate was covered by a mask (Supplementary Fig. 13a) and deposited Cr by vacuum evaporation. The substrate with 8 pixels was obtained (Supplementary Fig. 13b). The hemispherical photodetector for the location was fabricated by depositing the active material onto the substrate step by step. Supplementary Fig. 13c shows the circuit diagram of the read-out system of the hemispherical photodetector for location. Supplementary Fig.13d shows the printed circuit board (PCB) of the read-out system. Pixels were parallel connected while each pixel was connected with a resistance of 1 MΩ in series. The signals collected by the computer are the voltages of the resistance and are read out by NI9205. The optical photograph of the hemispherical photodetector and the read-out system are shown in Supplementary Fig. 13e. The bottom electrodes (x_1, x_2, …, x_8) were connected positive pole of the system by simple welding. The top electrode was lead out with a copper wire and connected to the negative electrode of the system.

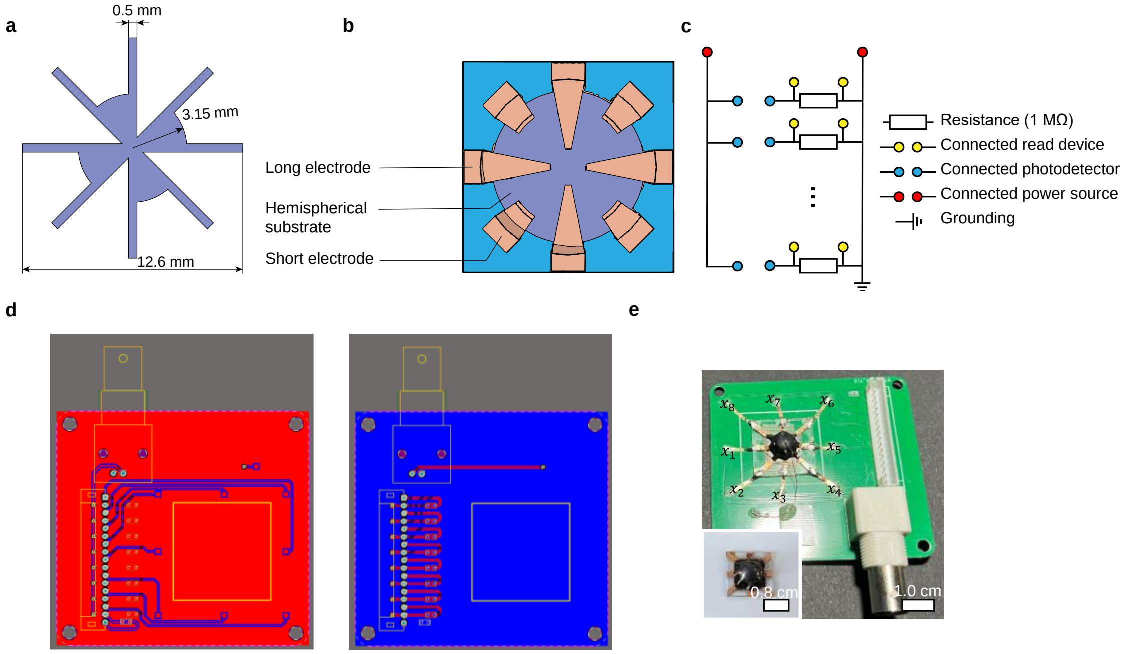

Supplementary Fig. 13 | The read-out system of the hemispherical photodetector for location. **a,** The shape of the mask to divide the electrode. **b,** The schematic diagram of the bottom electrodes of the hemispherical photodetector. **c,** The circuit diagram of the read-out system of the hemispherical photodetector for location. **d,** PCB of the read-out system of the hemispherical photodetector for location. Left: the top layer of the PCB. Right: the bottom layer of the PCB. **e,** The optical photograph of the hemispherical photodetector with the read-out PCB. Inset: The optical photograph of the hemispherical photodetector.

Supplementary Fig. 14a is the structure of Neural network fitting (NNF) for 2D location. The main algorithm of NNF is Bayesian regularization. The input data include 400 (20 × 20) observations with 8 features (from 8 pixels). The input data is obtained from the signals of the differential pixels when the light source is placed in a different position. The output data include 400 observations with 2 features (the relative coordinates (X, Y)) and are mentioned in the section on Artificial intelligence-assisted location. The relative coordinates of the light source are from (0, 0) to (20, 20). The distance between adjacent coordinate points is 3 mm. The correlation coefficient (R) of the training group, test group, and all groups are 0.99976, 0.99967, and 0.99976 respectively. Thus, using limited pixels with different shapes on the hemisphere for location is feasible. The analysis of the result of NNF for the location with color classification and spatial orientation was collected and shown in Supplementary Table 2, which also shows the analysis of the result of NNF for the location based on the planar photodetector.

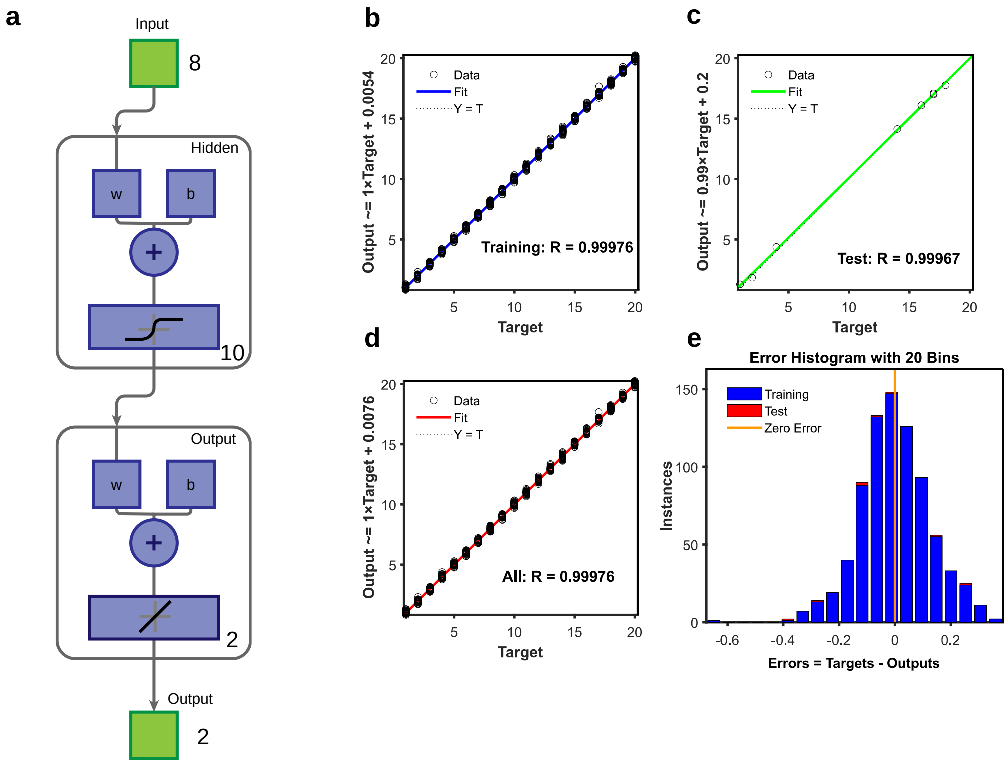

Supplementary Fig. 14 | The structure of the NNF and the analysis of the result from the NNF. a, The structure of the NNF. **b, c, d,** The performance of the model generated from the NNF. **e,** The error histogram of the result from the NNF in the training process.

Supplementary Table 2 | The structure of the NNF and the analysis of the result from the NNF.

	Input	Hidden layer	Output layer	R_{train}	R_{test}	R_{all}
2D trace	8	10	2	0.99976	0.99967	0.99976
2D trace (w color classification)	24	12	2	0.99954	0.99943	0.99953
3D trace	16	12	2	0.99978	0.99974	0.99978
2D trace (planar)	8	10	2	0.82381	0.74166	0.82339

To study the continuous process of location tracking, the position coordinates of the light source were recorded in chronological order and shown in Supplementary Fig. 15. The number of images is the numerical order of the image. Thus, the location of the object could be real-time. We also studied the planar photodetector for trace reconstruction. The result trace is unsuccessful and shown in Supplementary Fig. 15c. In Supplementary Fig. 15d, we present a schematic diagram illustrating the structure of the planar device employed for comparative analysis. Concurrently, we conducted measurements of the response matrixes corresponding to each pixel, as depicted in Supplementary Fig. 15e. Notably, their responses exhibit a high degree of similarity.

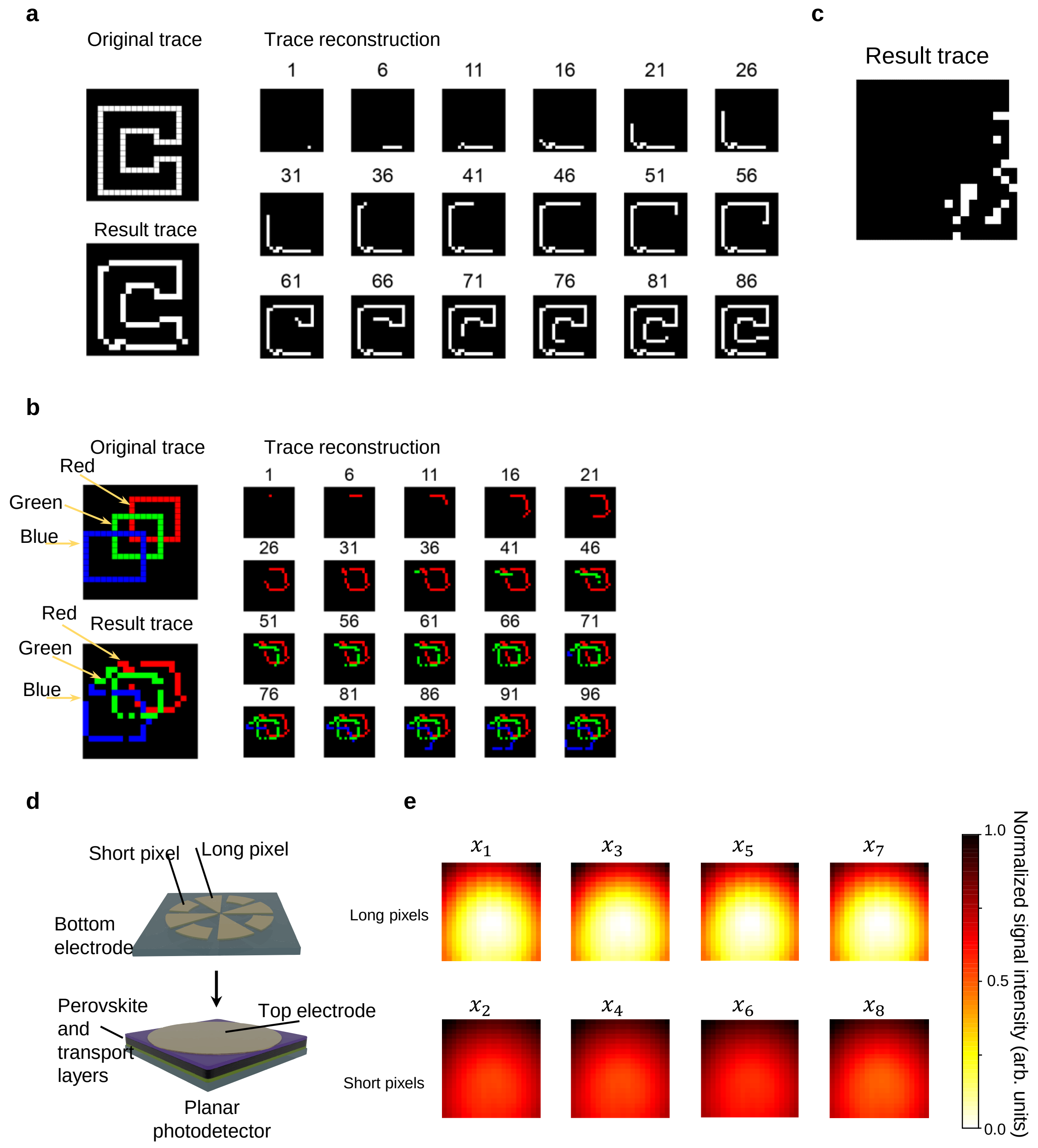

Supplementary Fig. 15 | Traces record of the NNF-assisted localization. a, Traces without color classification record of the NNF-assisted localization. **b,** Traces with color classification record of the NNF-assisted localization. **c,** The trace reconstruction executed by planar

photodetector. **d,** The scheme of the planar photodetector. **e,** The signal matrix of the differential pixels distributing different positions (x_1, x_2, …, x_8) of the planar photodetector.

Supplementary References

1. Lu Q, He W, Sun W, Feng Y, Zhan L, Luo Y. Synthesis of 2-arylamino-5-formyl-pyrimidines from the bis(hexafluorophosphate) Arnold salt. *J. Chem. Res.* **44**, 580-585 (2020).

2. Yoon H, *et al.* Miniaturized spectrometers with a tunable van der Waals junction. *Science* **378**, 296-299 (2022).

3. Jiang Y, *et al.* Synthesis-on-substrate of quantum dot solids. *Nature* **612**, 679-684 (2022).

4. Ma C, *et al.* Unveiling facet-dependent degradation and facet engineering for stable perovskite solar cells. *Science* **379**, 173-178 (2023).

5. Feng X, *et al.* Spray-coated perovskite hemispherical photodetector featuring narrow-band and wide-angle imaging. *Nat. Commun.* **13**, 6106 (2022).

6. Feng X, Tan M, Li M, Wei H, Yang B. Polyhydroxy Ester Stabilized Perovskite for Low Noise and Large Linear Dynamic Range of Self-Powered Photodetectors. *Nano Lett.* **21**, 1500-1507 (2021).

7. Lee B, Hwang T, Lee S, Shin B, Park B. Microstructural Evolution of Hybrid Perovskites Promoted by Chlorine and its Impact on the Performance of Solar Cell. *Sci. Rep.* **9**, 4803 (2019).
