# Supplementary material for: Differential perovskite hemispherical photodetector for intelligent imaging and location tracking
Source: Nat Commun. 2024 Jan 17;15:577. doi: 10.1038/s41467-024-44857-4 (PMC10794423; doi:10.1038/s41467-024-44857-4)
Supplement: Supplementary file 3 — Description of Additional Supplementary Files [file 41467_2024_44857_MOESM3_ESM.pdf]

Description of Additional Supplementary Files

Supplementary Movie 1 | The motion trajectory animation of the light source is obtained through the application of trajectory reconstruction algorithms. The animation is generated by integrating the positional coordinates of the light source at each time point.

Supplementary Movie 2 | The motion trajectory and color-coded animation of the light source are obtained through a trajectory reconstruction algorithm incorporating color classification. The animation is generated by integrating the positional coordinates of the light source at each time point, with colors representing red, green, and blue.

Supplementary Movie 3 | The motion trajectory animation of the light source is obtained through a spatial trajectory reconstruction algorithm. The animation is generated by integrating the positional coordinates of the light source at each time point.

Supplementary Software 1 | MATLAB code for image color classification imaging and MATLAB code for the reconstruction of light source position and motion trajectory for a hemispherical photodetector. The reconstruction of light source position and motion trajectory is categorized into three types: 1. without color recognition, involving planar reconstruction; 2. with color recognition, also employing planar reconstruction; 3. without color recognition, utilizing spatial reconstruction.
